# Mixed trichuroid infestation in a dog from Italy

**DOI:** 10.1186/1756-3305-5-128

**Published:** 2012-06-25

**Authors:** Angela Di Cesare, Giuseppe Castagna, Silvana Meloni, Domenico Otranto, Donato Traversa

**Affiliations:** 1 Department of Comparative Biomedical Sciences, University of Teramo, Valenzano, Italy; 2 Department of Public Health and Zootechny, University of Bari, Valenzano, Bari, Italy

**Keywords:** *Capillaria aerophila*, *Capillaria boehmi*, *Trichuris vulpis*, Italy, Mixed infestation, Diagnosis

## Abstract

**Background:**

*Capillaria aerophila*, *Capillaria boehmi* and *Trichuris vulpis* are trichuroid nematodes affecting wild and companion animals all over the World. The canine intestinal whipworm, *T. vulpis*, is the most common and well- known in veterinary practice, whereas the respiratory *C. aerophila* and *C. boehmi* have been rarely reported in pets as a likely consequence of overlapping morphometric and morphological features of the eggs, which impair a correct etiological diagnosis.

**Findings:**

In December 2011, a mixed infestation by *T. vulpis*, *C. aerophila* and *C. boehmi* was diagnosed in an asymptomatic dog living in central Italy. Morphometric and morphological findings and pictures of the eggs found at the copromicroscopic analysis are herein reported.

**Conclusions:**

The present work demonstrates that when trichuroid eggs are found in a faecal sample from a dog, a careful morphological and morphometric analysis of individual parasite elements is mandatory. Key diagnostic features (i.e., size, wall surface pattern and aspects of plugs) should be carefully examined when eggs with overlapping shape and appearance are detected. In conclusion, given the importance in clinical practice of canine trichuroids and the zoonotic potential of *C. aerophila*, these nematodes should be included into the differential diagnosis of intestinal and respiratory parasitoses of dogs by a thorough microscopic analysis of all trichuroid ova present in microscopic fields.

## Findings

### Background

Parasitic nematodes belonging to the family Trichuridae infest wild and domestic animals, and human beings as well. The so-called “canine whipworm” *Trichuris vulpis* is the most known and it affects the large bowel of dogs and wild canids. The life cycle of *T. vulpis*, which is direct and based on a soil-transmitted oral route of infestation *via* embryonated eggs, is well understood, albeit some biological aspects of this nematode (e.g., blood-sucking and zoonotic abilities) have yet to be elucidated [1]. Conversely, the respiratory nematode *Capillaria aerophila* is poorly known. Adult stages inhabit the epithelium of bronchioles, bronchi and trachea of foxes, badgers, raccoons, bears, dogs, cats and, occasionally, humans [2-4]. The life cycle of *C. aerophila* is direct and animals become infected by ingesting environmental embryonated eggs; earthworms can be implicated in the transmission of *C. aerophila*, although whether as facultative intermediate or paratenic hosts is yet to be understood [2,5]. A third species, *Capillaria boehmi*, lives in the nasal cavities and sinuses of wild canids (e.g., foxes and wolves) and domestic dogs, and its life cycle is unknown [6].

While the canine whipworm is a ubiquitous parasite infesting all categories of dogs (i.e. pets, kenneled and stray dogs), with high prevalence all over the World [1], *C. aerophila* and *C. boehmi* are commonly found in wildlife [6]. Nonetheless, in the last decade an increasing trend in the number of infestations in domestic animals has been recorded, being *C. aerophila* reported in dogs from Europe, North America and Australia (rev. in Traversa *et al*., 2010), and *C. boehmi* in dogs from Europe and North America [7-10]. Most infestations by canine whipworms in adult patients can be asymptomatic, although heavy parasitic burdens may induce colitis with different symptoms irrespective of the age of infected animals [1]. Animals harboring *C. aerophila* can be either asymptomatic or display different respiratory distresses with varying degree of severity [2,11]. Dogs infected with *C. boehmi* may be asymptomatic but, when symptoms occur, infected animals show upper respiratory tract signs [12,13].

The diagnosis of these trichuroid infestations relies on the detection of the typical eggs through standard fecal floatations irrespective of the great morphological similarities of the ova, which pose important challenges in epidemiological and clinical settings.

This article reports the simultaneous occurrence of *T. vulpis*, *C. aerophila* and *C. boehmi* in a dog and presents key morphological characters for a reliable diagnosis at the species level.

### Methods

In December 2011 a dog living in Latina Municipality (Lazio region, Central Italy) was clinically examined and subjected to a copromicroscopic analysis. A faecal sample collected from the rectum of the dog was examined at the Parasitology Laboratory of the Department of Comparative Biomedical Science of the University of Teramo using a standard floatation procedure incorporating a zinc sulphate solution with a specific gravity of 1.350 [14]. Briefly, about five grams of faeces were added to 20 ml of floatation solution and then the mixture was strained through a double layer of cheesecloth. The solution was poured into a 15-ml centrifuge tube and centrifuged at 1200 rpm for five minutes. A pasteur pipette was then used to transfer a supernatant aliquot of ~100 μl to a glass slide, and the slide was examined using a light microscope (Axioscope 40, Zeiss, Oberkochen, Germany) at 200X and 400X magnifications [14].

Given that several trichuroid eggs (i.e. n = 45) with overlapping shape and appearance, but with slight differences, were detected, all parasitic elements found in the examined slide (coverslip of 24x50 mm) were undertaken and a thorough morphological and morphometric analysis were carried out.

### Results

**Figure 1:**
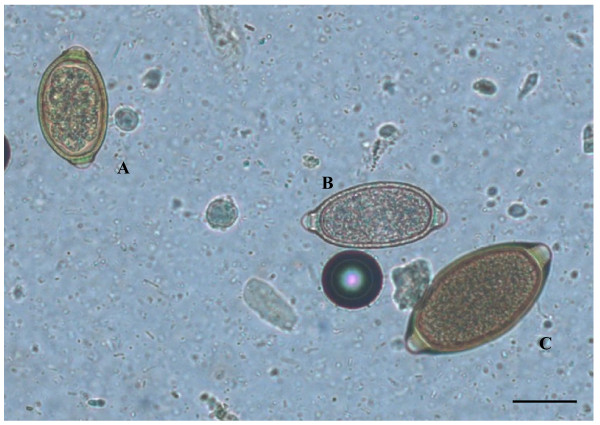
**Eggs of*Capillaria boehmi*(A),*Capillaria aerophila*(B) and*Trichuris vulpis*(C) detected by light microscopy examination (200x magnification).** Scale bar = 30 μm.

**Figure 2:**
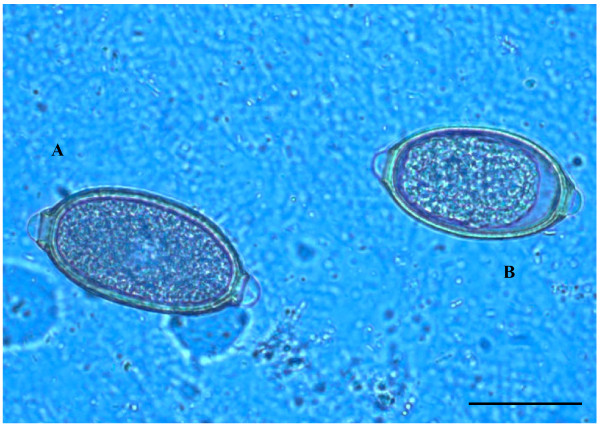
**Eggs of*Capillaria aerophila*(A) and*Capillaria boehmi*(B) detected by light microscopy examination (400x magnification)**. Scale bar = 30 μm.

**Figure 3:**
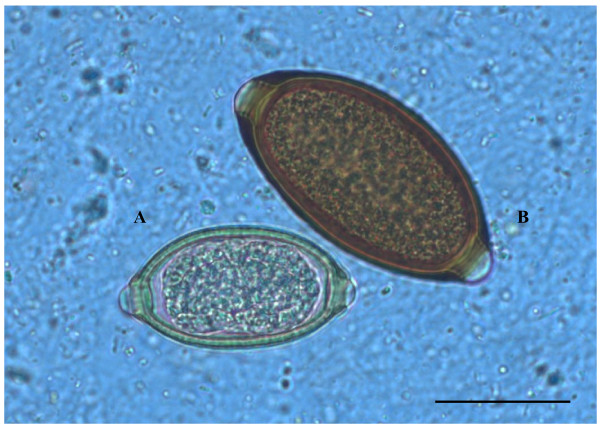
**Eggs of*Capillaria boehmi*(A) and*Trichuris vulpis*(B) detected by light microscopy examination (400x magnification).** Scale bar = 30 μm.

The dog presented no clinical signs and was apparently healthy. All eggs in the sample showed a lemon/barrel-like appearance with two plugs at the extremities. However, the morphologic and morphometric examination, conducted using diagnostic keys (Table 1), showed the presence of three different trichuroids simultaneously affecting the dog, i.e. *T. vulpis* (n = 27 eggs), *C. aerophila* (n = 12 eggs) and *C. boehmi* (n = 7 eggs) (Figures 1, 2, 3).

**Table 1:** **Differential features of eggs of*Trichuris vulpis*,*Capillaria aerophila*and*Capillaria boehmi***

**Nematode**	**Length μm**	**Width μm**	**Morphological features**	**Plugs**	**Shell**	**Reference**
*Trichuris vulpis*	72-94	31-42	lemon-like shape, brownish	symmetrical presence of rings	thick and smooth wall	[1,5]
*Capillaria aerophila*	60-65	25-40	barrel-like shape, zygote fills the egg	asymmetrical no rings	striated outer shell with a network of anastomosis ridges and bridges	[4,5]
*Capillaria boehmi*	50-60	30-35	barrel-like shape, clear to golden, space between the embryo and the wall	asymmetrical no rings	tiny pits on the surface of the wall	[4,10]

**Figure 4:**
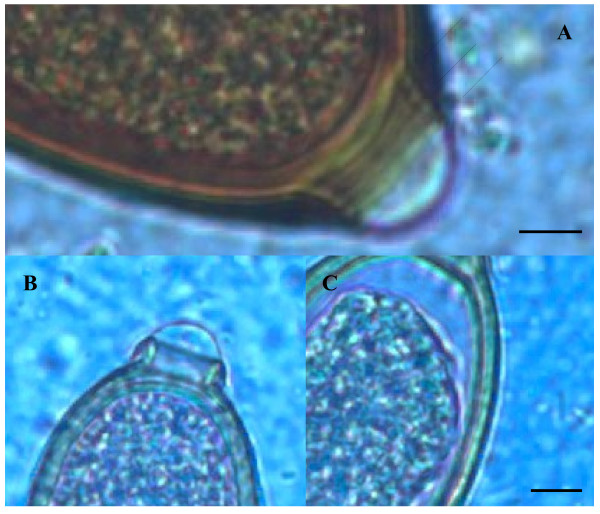
**Egg polar plugs of*Trichuris vulpis*(A),*Capillaria aerophila*(B) and*Capillaria boehmi*(C).** Arrows indicate thickening at the base of the bipolar plug of *T. vulpis*, which are absent in *C. aerophila* and *C. boehmi*. Scale bars = 5 μm.

**Figure 5:**
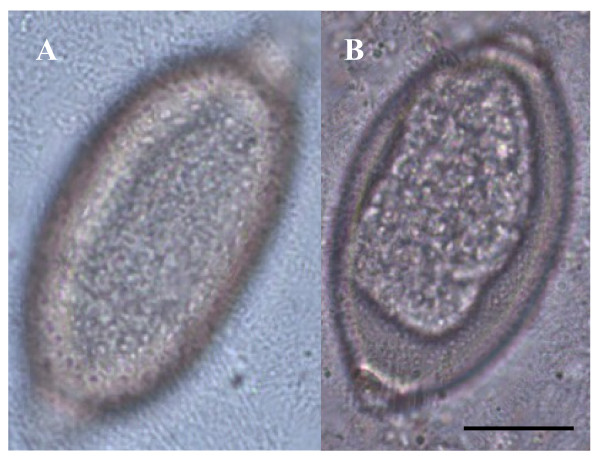
**Outer shell of the egg of*Capillaria aerophila*(A) and*Capillaria boehmi*(B) showing the typical network of anastomosing ridges and bridges and the presence of tiny pits, respectively**. Scale bar = 15 μm.

Ova of *T. vulpis* were symmetrical, with ring-like thickening at the plug bases (Figure 4) and presented a brown color, smooth surface and a size ranging from 83.50 ± 3.00 μm in length and 36.00 ± 3.50 μm in width. *Capillaria aerophila* eggs were smaller than those of *T. vulpis*, i.e. lengths of major and minor axes were 64.45 ± 1.50 μm and 34.95 ± 3.40 μm, respectively. Also, they showed asymmetrical bipolar plugs with no ring thickening (Figure 4) and a network of anastomosing ridges and bridges on their walls (Figure 5). Eggs of *C. boehmi* were morphologically kindred to those of *C. aerophila*, having asymmetry of no-ringed plugs (Figure 4) and being smaller than those of *T. vulpis*. However, these eggs were even smaller than those of *C. aerophila*, with a size of 55.30 ± 1.30 μm in length and 32.40 ± 2.60 μm in width, and presented a surface characterized by several tiny pits and a space between embryo and wall (Figure 5).

### Discussion and conclusions

This study reports for the first time an infestation by three species of trichuroids in the same animal, thus indicating that besides the most commonly retrieved *T. vulpis,* also *C. aerophila* and *C. boehmi* may infect dogs simultaneously. Thus, a thorough and careful microscopic examination of trichuroid eggs in faeces of dogs is always mandatory when barrel-shaped or lemon-like eggs are found.

Although nematodes ranked within the *Capillaria* genus occur in several geographic areas [4,6,10,11,15] these species are neglected as a likely effect of the drawbacks in copromicroscopical identification of their eggs, which are often misdiagnosed with those of *T. vulpis*[5]. Additionally, eggs of *C. boehmi* can be mistaken with those of *C. aerophila*. In fact, while lung capillariosis has been frequently reported in dogs in the past few years, this is not the case for canine nasal capillariosis. As an example, the presence of *C. boehmi* in Italy is rather anecdotal, with only one report published [8] and recent personal communications (Veronesi, 2010, Perrucci 2012, pers. comm. to DT). The risk of misidentification is high and this is even more so in the presence of mixed infestations in which one species is more prevalent over the other/s, as in the case presented herein. In fact, if in the present report an analyst would have taken into account only more prevalent eggs without a careful examination of the whole microscopic field, the dogs would have been diagnosed only with a whipworm infestation.

An accurate appraisal of trichuroid eggs is of importance from a clinical standpoint. Dogs infected by *T. vulpis* may suffer from relevant systemic (e.g. reduced growth rate, wasting, weight loss, lethargy, anemia) and intestinal distresses (e.g. watery and haemorrhagic diarrhoea), which overlap a plethora of intestinal diseases with multiple etiology [1]. Also respiratory capillariosis induces aspecific symptoms, e.g. nasal discharge, sneezing, wheezing, chronic cough, dyspnea (*C. aerophila*) or sneezing, mucopurulent and/or bloody nasal scolum and anosmia (*C. boehmi*) [4,5,9].

On the other hand, the presence of asymptomatic animals may be frequent [1,4,8] and the lack of clinical signs in the dog herein examined confirms such evidence.

Hence, the lack of specificity of clinical signs or even the absence of symptoms in intestinal trichurosis and respiratory capillarioses, may complicate the work of veterinarians in diagnosing these parasitoses in pets.

A correct diagnosis of trichuroid infestations is pivotal considering a potential zoonotic role. In fact, while *C. boehmi* does not infest humans, the role of *T. vulpi*s as a zoonotic agent is still controversial, and *C. aerophila* induces, although seldom, pulmonary capillariosis in people, causing fever, bronchitis, coughing, haemoptisis, dyspnoea and may mimic bronchial carcinoma [1,3,4].

Further molecular studies are warranted to support a reliable identification of *T. vulpi*s, *C. aerophila* and *C. boehmi* eggs in faecal samples. Recently, the efficiency of a mitochondrial DNA based assay in the diagnosis of lung capillariosis by *C. aerophila* has been demonstrated [16]. Similar studies are also presently ongoing for *T. vulpi*s and *C. boehmi*, with the aim of validating a genetic tool for the simultaneous differentiation of canid trichuroids (Di Cesare and Traversa, unpublished).

In conclusion, the guard against these three trichuroids affecting dogs should be kept high and they should always be included in the differential diagnosis of intestinal and respiratory diseases of companion animals. Veterinarians, parasitologists and laboratory technicians should be aware of their occurrence and always perform a thorough microscopic analysis of all trichuroid ova present in microscopic fields when examining any faecal sample collected from a dog.

## Competing interests

The authors declare that they have no competing interests.

## Authors’ contributions

ADC: contributed in laboratory examinations and morphological and morphometric tests, interpretation of data and drafted the manuscript. GC: participated in morphological and morphometric tests and image analysis and elaboration. SM: examined the dog, collected the sample and performed morphological and morphometric analysis. DO: participated in analysis and interpretation of data and thoroughly commented on the manuscript. DT: participated in analysis and interpretation of data, and conceived and edited the manuscript. All authors reviewed and approved the manuscript.

## References

[B1] Traversa D Are we paying too much attention to cardio-pulmonary nematodes and neglecting old-fashioned worms like Trichuris vulpis? Parasit Vectors 2011 8 4 32 10.1186/1756-3305-4-32 PMC3063211 21385441

[B2] Taylor MA Coop RL Wall RL Veterinary Parasitology 2007 3 Blackwell Publishing, Oxford, UK

[B3] Lalosević D Lalosević V Klem I Stanojev-Jovanović D Pozio E Pulmonary capillariasis miming bronchial carcinoma AmJTrop Med Hyg 2008 78 14 16 18187778

[B4] Traversa D Di Cesare A Conboy G Canine and feline cardiopulmonary parasitic nematodes in Europe: emerging and underestimated Parasit Vectors 2010 23 3 62 10.1186/1756-3305-3-62 PMC2923136 20653938

[B5] Traversa D Di Cesare A Lia RP Castagna G Meloni S Heine J Strube K Milillo P Otranto D Meckes O Schaper R New insights into morphological and biological features of Capillaria aerophila (Trichocephalida, Trichuridae) Parasitol Res 2011 109 Suppl 1 S97 S104 21739379 10.1007/s00436-011-2406-4

[B6] Conboy GA Helminth parasites of the canine and feline respiratory tract Vet Clin N Am Small Anim Pract 2009 39 1109 1126 10.1016/j.cvsm.2009.06.006 19932366

[B7] Gajewska A Gorski P Kotomski G Bogdanowicz M Klockiewicz M Kazimierczak K Changes in parasites of dogs and cats from Warsaw and suburbs during the period of 1974-2002. Part III. Roundworms Zycie Weterynaryjne 2004 79 208 212 In Polish

[B8] De Liberato C Mazzanti S Scaramozzino P First report of Eucoleus bohmi (Nematoda Trichuridea) from Italy: parasitological findings and veterinary implications Parassitologia 2009 51 43 45

[B9] Piperisova I Neel JA Tarigo J What is your diagnosis? Nasal discharge from a dog Vet Clin Pathol 2010 39 121 122 10.1111/j.1939-165X.2009.00174.x 19650779

[B10] Baan M Kidder AC Johnson SE Sherding RG Rhinoscopic Diagnosis of Eucoleus boehmi Infection in a Dog J Am Anim Hosp Assoc 2011 47 60 63 10.5326/JAAHA-MS-5707 21164166

[B11] Traversa D Di Cesare A Milillo P Iorio R Otranto D Infection by Eucoleus aerophilus in dogs and cats: is another extra-intestinal parasitic nematode of pets emerging in Italy? Res Vet Sci 2009 87 2 270 272 10.1016/j.rvsc.2009.02.006 19298989

[B12] Evinger JV Kazacos KR Cantwell HD Ivermectin for treatment of nasal capillariasis in dog J Am Vet Med Assoc 1985 186 174 175 3838298

[B13] Campbell BG Little MB Identification of eggs of a nematode (Eucoleus boehmi) from the nasal mucosa of North American dogs J Am Vet Med Assoc 1991 54 145 154 2061172

[B14] Sloss MW Kemp RL Zajac AM Veterinary Clinical Parasitology 1994 Iowa State University Press, Ames, IA

[B15] Di Cesare A Castagna G Meloni S Milillo P Latrofa MS Otranto D Traversa D Canine and feline infections by cardiopulmonary nematodes in central and southern Italy Parasitol Res 2011 109 Suppl 1 S87 S96 21739378 10.1007/s00436-011-2405-5

[B16] Di Cesare A Castagna G Otranto D Meloni S Milillo P Latrofa MS Paoletti B Bartolini R Traversa D Molecular diagnosis of Capillaria aerophila, an agent of canine and feline pulmonary capillariosis J Clin Microbiol 2012 50 1958 1963 in press 10.1128/JCM.00103-12 22442326 PMC3372154

